# Post-radiation Uterine Carcinosarcoma: A Case Report

**DOI:** 10.7759/cureus.116167

**Published:** 2026-09-13

**Authors:** Inês Dias, Clara Casanova, Teresa Margarida Cunha

**Affiliations:** 1 Radiology, Instituto Português de Oncologia de Lisboa Francisco Gentil, Lisbon, PRT; 2 Radiology, Unidade Local de Saúde de Gaia e Espinho, Vila Nova de Gaia, PRT

**Keywords:** cervical carcinoma, hematometra, pelvic radiotherapy, radiation-associated endometrial cancer, uterine carcinosarcoma

## Abstract

Uterine carcinosarcoma is a rare, malignant tumor of the endometrium composed of both high-grade epithelial (carcinomatous) and mesenchymal (sarcomatous) components, representing one of the most aggressive histological subtypes of endometrial carcinoma. Prior pelvic radiotherapy has been associated with an increased risk of subsequent aggressive endometrial malignancies, although the latency between irradiation and tumor development may vary considerably. Diagnosis may be delayed because early symptoms can mimic benign post-radiation sequelae.

We report a woman in her 60s who, seven years after chemoradiotherapy for cervical squamous cell carcinoma, developed a three-month history of intermittent pelvic pain and mild vaginal bleeding. Pelvic MRI showed marked hematometra and a polypoid endometrial tumor invading the outer half of the myometrium. Histology after hysterectomy confirmed uterine carcinosarcoma with heterologous sarcomatous elements. Six months after diagnosis, biopsy-confirmed pleural metastases developed, and palliative chemotherapy was initiated.

This case highlights hematometra as a nonspecific but potentially concerning radiological finding when associated with a suspicious endometrial mass in a previously irradiated patient, emphasizing the importance of timely investigation of new pelvic symptoms.

## Introduction

Uterine carcinosarcoma is a rare, malignant tumor of the endometrium composed of both high-grade epithelial (carcinomatous) and mesenchymal (sarcomatous) components, representing a highly aggressive histologic subtype of endometrial tumor [1,2]. Prior pelvic radiotherapy, particularly for cervical carcinoma, has been associated with an increased risk of subsequent aggressive, poorly differentiated endometrial malignancies, including carcinosarcoma, which occur more frequently after irradiation than sporadically [3-5]. Radiation-related tissue changes, including cervical stenosis, may impair uterine drainage and lead to hematometra, defined as the accumulation of blood within the uterine cavity.

The latency between radiation exposure and the development of a subsequent malignancy can vary considerably, with reported cases occurring several years after irradiation. Presentation may be delayed because symptoms such as pelvic pain, bleeding, and uterine distension can mimic benign post-radiation sequelae, such as cervical stenosis with hematometra [3,4].

This report describes a rare case of uterine carcinosarcoma diagnosed seven years after chemoradiotherapy for cervical carcinoma and discusses the clinical and imaging features, particularly hematometra as a nonspecific but potentially concerning radiological finding when associated with a suspicious endometrial mass in a previously irradiated patient, emphasizing the importance of timely investigation of new pelvic symptoms.

## Case presentation

A woman in her 60s, who was postmenopausal, had previously been treated with chemoradiotherapy for FIGO 2018 stage IIA1 squamous cell carcinoma of the cervix seven years earlier (Figure 1) and had remained free of documented recurrence during this interval. Seven years after completion of treatment, she presented with a three-month history of intermittent pelvic pain and mild vaginal bleeding, prompting gynecological evaluation. Examination revealed uterine enlargement without a palpable mass and marked cervical stenosis. Cervical cytology was negative for intraepithelial lesion or malignancy and showed moderate epithelial atrophy. CA-125 was elevated at 130 U/mL. Given the history of previous pelvic irradiation, marked cervical stenosis, and new pelvic symptoms, pelvic MRI was performed to further investigate the uterine enlargement and possible impaired uterine drainage.

**Figure 1:**
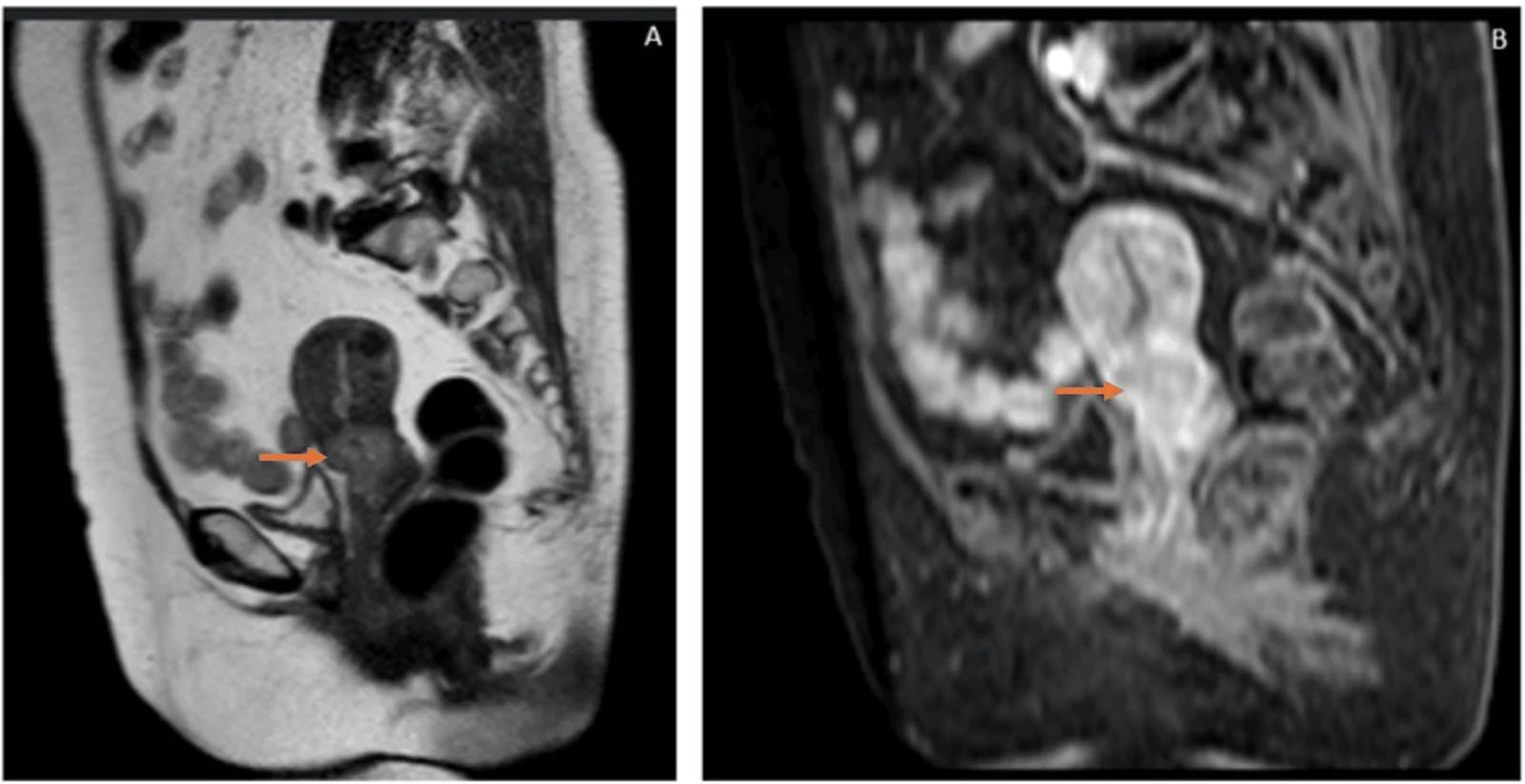
Abdominal and pelvic MRI at the time of diagnosis of the patient’s prior cervical tumor. (A) Sagittal T2-weighted image and (B) post-contrast image showing the patient’s original squamous cell carcinoma of the cervix (orange arrows), diagnosed seven years before the subsequent uterine carcinosarcoma. Histopathological diagnosis: well-differentiated squamous cell carcinoma of the cervix, FIGO stage IIA1. This historical imaging is provided to document the primary cervical malignancy for which the patient subsequently underwent pelvic chemoradiotherapy, preceding the development of the radiation-associated uterine carcinosarcoma.

Pelvic MRI showed marked hematometra, with the endometrial cavity distended to approximately 840 mL on MRI, and a polypoid endometrial tumor measuring 3.1 × 2.7 × 3 cm (longitudinal × transversal × anteroposterior), invading the outer half of the myometrium (Figures 2-3). The tumor was isointense to the myometrium on T1-weighted imaging and showed heterogeneous signal intensity on T2-weighted imaging, predominantly higher than that of the myometrium, with restricted diffusion and heterogeneous post-contrast enhancement. The presence of a solid polypoid mass with restricted diffusion, heterogeneous enhancement, and myometrial invasion raised suspicion for an underlying endometrial malignancy rather than hematometra alone. There was no extension to the cervical stroma, parametrium, bladder, or rectum, and no pelvic or para-aortic lymphadenopathy.

**Figure 2:**
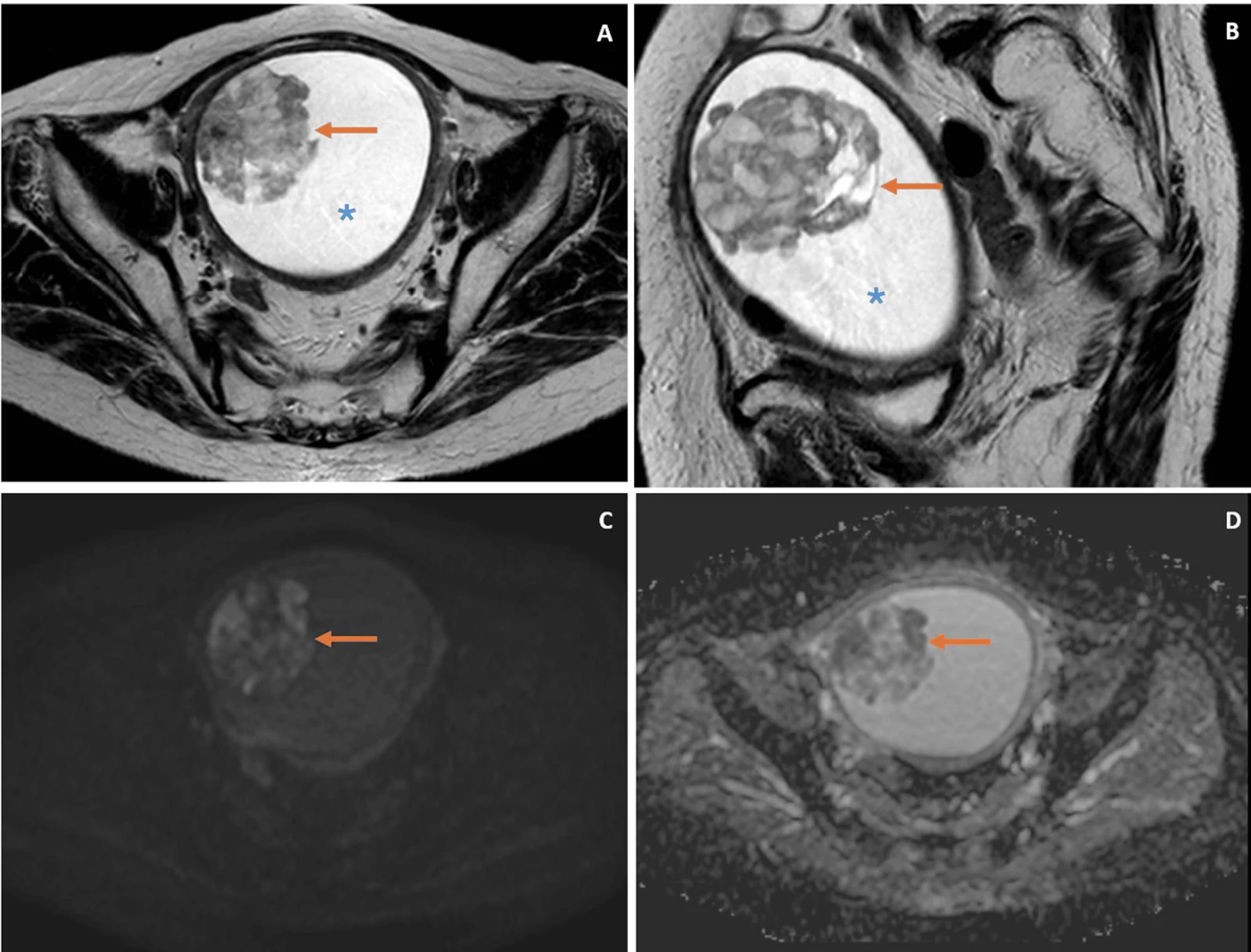
Abdominal and pelvic MRI showing hematometra and an underlying malignant polypoid endometrial tumor. A markedly distended endometrial cavity containing hematometra (blue asterisk; approximately 840 mL) is seen surrounding the solid polypoid endometrial tumor (orange arrows). The tumor demonstrates heterogeneous signal intensity, predominantly higher than that of the myometrium, on (A) axial and (B) sagittal T2-weighted imaging. (C) Diffusion-weighted imaging with a b-value of 1000 s/mm² (b1000) demonstrates restricted diffusion within the tumor, with corresponding low signal intensity on the (D) apparent diffusion coefficient (ADC) map.

**Figure 3:**
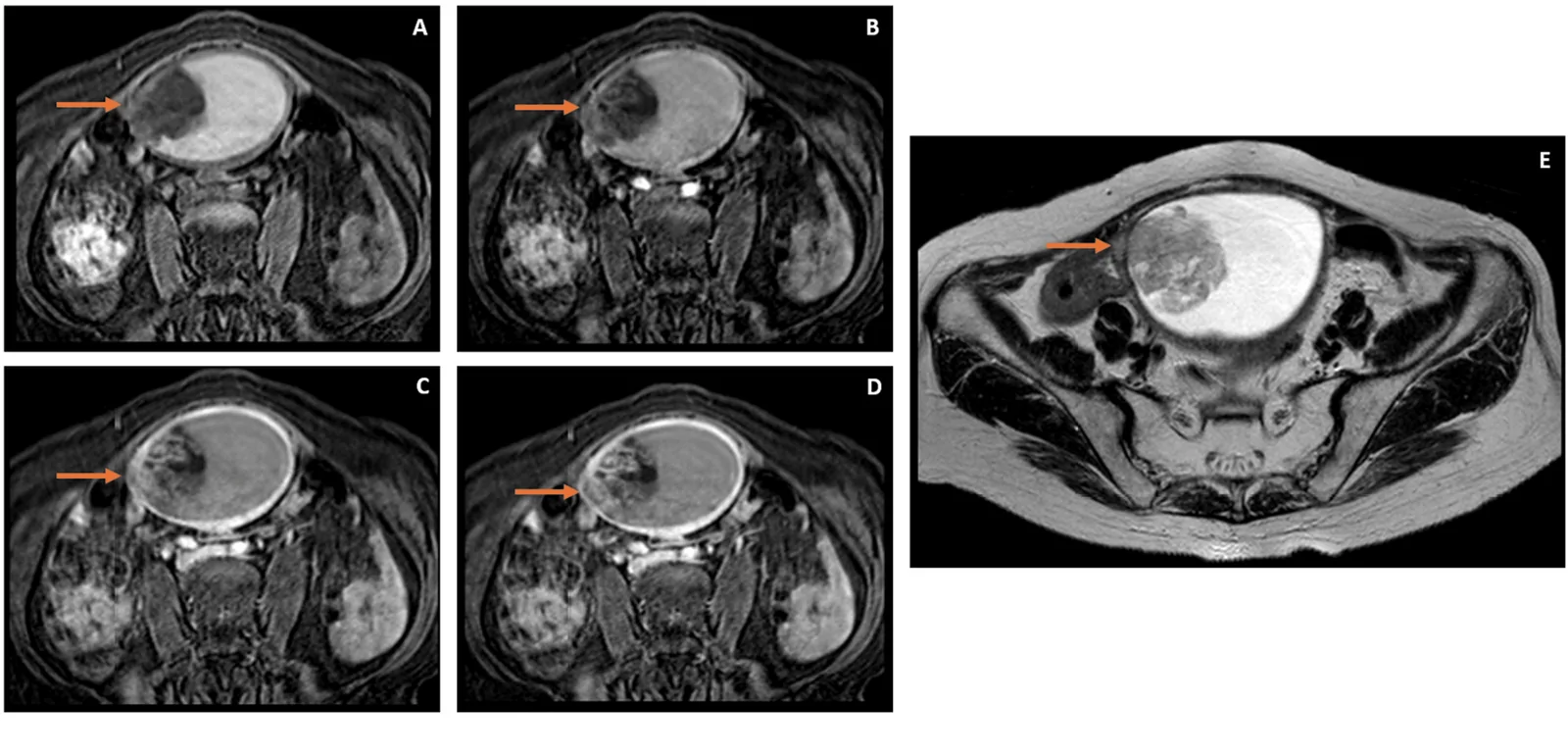
Abdominal and pelvic MRI demonstrating myometrial invasion by the endometrial tumor. (A-D) Dynamic contrast-enhanced MRI before and after intravenous gadolinium administration and (E) axial T2-weighted image demonstrating heterogeneous enhancement and extension into the outer half of the myometrium (orange arrows), consistent with deep myometrial invasion.

Total hysterectomy with bilateral salpingo-oophorectomy was performed. Sentinel lymph node assessment and pelvic or para-aortic lymph node sampling were not documented in the available surgical or pathological records. Histology confirmed uterine carcinosarcoma (Figure 4), with an epithelial component showing endometrioid, serous, squamous, and undifferentiated areas and a mesenchymal component with heterologous rhabdomyosarcoma, liposarcoma, and chondrosarcoma. Myometrial invasion and lymphovascular space invasion were present, and the tumor was classified as FIGO 2023 IIC.

**Figure 4:**
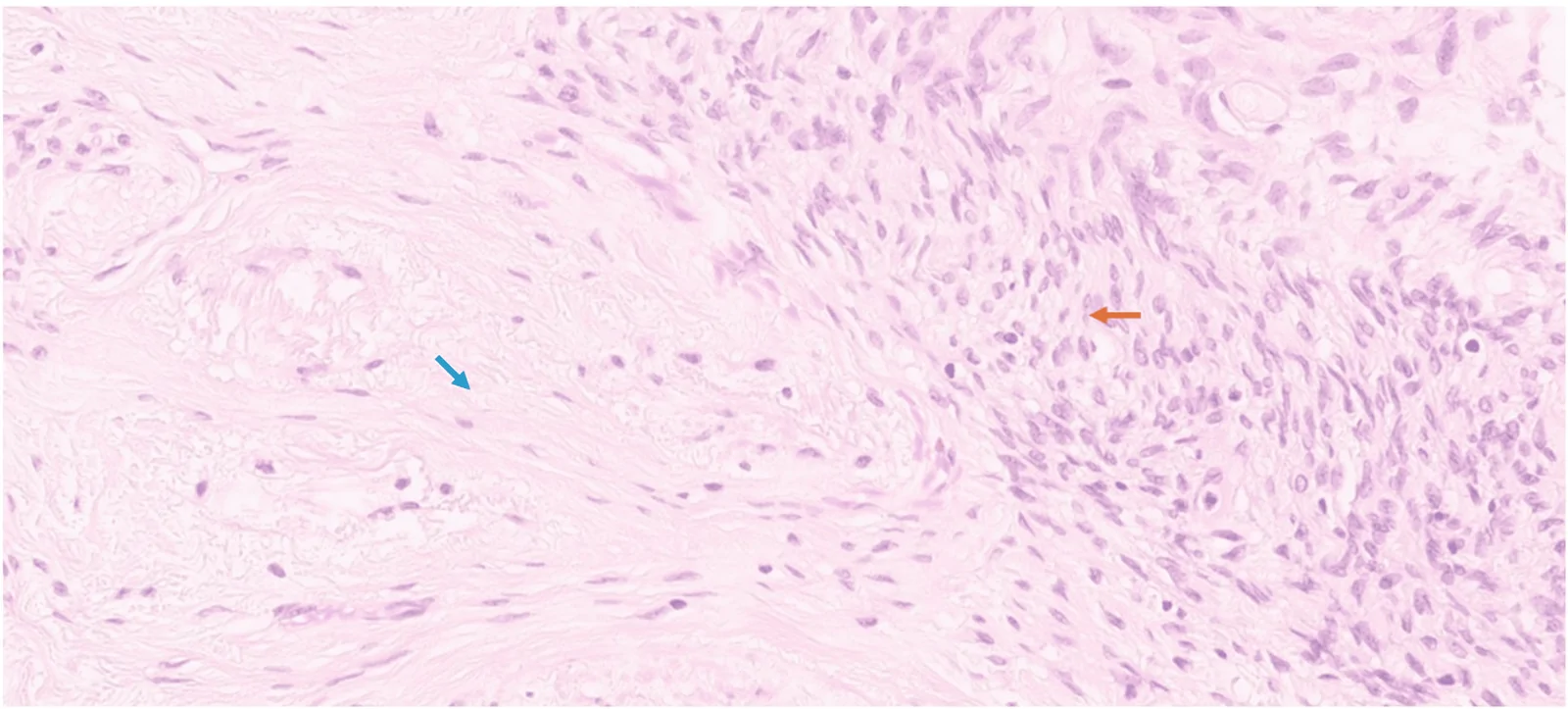
Histopathological examination of uterine carcinosarcoma. The carcinomatous epithelial component on the right (orange arrow) and the sarcomatous mesenchymal component, containing heterologous elements, on the left (blue arrow) are shown (hematoxylin and eosin stain, magnification ×20).

Following surgery, the patient initiated adjuvant chemotherapy; however, treatment was discontinued during the first cycle due to treatment-related adverse effects, and no further adjuvant chemotherapy was administered. Six months after diagnosis, the patient's clinical condition worsened. Thoracic, abdominal, and pelvic CT showed a moderate right pleural effusion with pleural metastatic deposits, confirmed on biopsy as metastatic uterine carcinosarcoma. Palliative chemotherapy was subsequently initiated. At the time of the last available follow-up, the patient remained under palliative treatment and had not yet undergone repeat imaging to assess treatment response.

## Discussion

Uterine carcinosarcoma is a rare mixed epithelial and mesenchymal tumor and one of the most aggressive histological subtypes of endometrial cancer, typically presenting with postmenopausal bleeding [1,2]. In our patient, presentation was instead dominated by intermittent pelvic pain and mild vaginal bleeding in the setting of marked hematometra.

Pelvic radiotherapy for cervical carcinoma has been associated with a higher long-term risk of poorly differentiated, aggressive endometrial cancer subtypes, including carcinosarcoma, which occur more frequently after irradiation than in the sporadic setting [3-5]. The seven-year interval observed in our case is consistent with latency intervals reported for radiation-associated endometrial malignancies, although these intervals may vary. Given the prior pelvic radiotherapy and the absence of documented recurrent cervical cancer during the intervening period, the uterine carcinosarcoma was considered a subsequent uterine primary rather than a recurrence of the original cervical carcinoma. However, a causal relationship with radiotherapy cannot be established from this single case.

Hematometra may occur in previously irradiated patients, particularly in the presence of cervical stenosis, but it is a nonspecific finding with several possible causes. In our patient, marked cervical stenosis was documented on gynecological examination, while the contribution of tumor-related hemorrhage or obstruction to the hematometra could not be definitively established. MRI was central to this case, identifying both the hematometra and the underlying polypoid tumor, with diffusion restriction, heterogeneous enhancement, and myometrial invasion raising suspicion for an underlying malignancy and prompting definitive surgical management.

Uterine carcinosarcoma is associated with a high risk of recurrence, and treatment options for recurrent or metastatic disease remain limited, with generally poor outcomes [2]. This is consistent with the aggressive clinical course observed in our patient, who developed biopsy-confirmed pleural metastases six months after diagnosis.

This case reinforces the need for a high index of suspicion for a subsequent uterine malignancy in patients with prior pelvic radiotherapy who present with new pelvic symptoms, even many years after treatment. Hematometra should not be regarded as specific for carcinosarcoma, but when associated with a solid endometrial mass, restricted diffusion, heterogeneous enhancement, or myometrial invasion, it may represent a nonspecific but potentially concerning radiological clue warranting further investigation.

## Conclusions

Uterine carcinosarcoma is a rare but aggressive malignancy that can occur years after pelvic radiotherapy, including radiotherapy for cervical cancer. In the present case, the seven-year interval and absence of documented recurrent cervical cancer support consideration of a subsequent uterine primary, although a causal relationship between radiotherapy and carcinosarcoma cannot be established. Hematometra is a nonspecific finding but may represent a potentially concerning radiological clue to an underlying endometrial malignancy in previously irradiated patients, particularly when associated with a solid endometrial mass and suspicious MRI features. Although this single case cannot establish the predictive value of hematometra or demonstrate the benefit of long-term surveillance, it supports continued clinical vigilance and is consistent with the rationale for long-term gynecological follow-up described in the existing literature. New pelvic symptoms in patients with a history of pelvic radiotherapy should prompt timely clinical and imaging evaluation.
